# Morphine Plus Bupivacaine *Vs.* Morphine Peridural Analgesia in Abdominal Surgery: The Effects on Postoperative Course in Major Hepatobiliary Surgery

**DOI:** 10.1155/2000/64016

**Published:** 2000-08

**Authors:** G. Barzoi, S. Carluccio, B. Bianchi, S. Vassia, G. Colucci, G. L. Mangiante

**Affiliations:** 1 Institute of Anesthesiology and Intensive Care University of Verona Italy; 2 Department of Surgery University of Verona Italy; 3 Department of Surgery University of Verona Via delle Menegone, 10 Verona 37134 Italy

## Abstract

Anaesthesia and surgical procedures lead to a reduction of intestinal motility, and opioids may produce a postoperative ileus, that might delay postoperative feeding. The aim of this prospective randomised study is to test whether or not different kinds of epidural analgesia (Group A: morphine 0.00 17 mg/kg/h and bupivacaine 0.125% – 0.058 mg/kg/h; Group B: morphine alone 0.035mg/kg/12h in the postoperative period) allow earlier postoperative enteral feeding, enhance intestinal motility a passage of flatus and help avoid complications, such as nausea, vomiting, ileus, diarrhoea, pneumonia or other infective diseases. We included in the study 60 patients (28 males and 32 females) with a mean age of 61.2 years (range 50–70) and with an ASA score of 2 or 3. All patients had hepato–biliary-pancreatic neoplasm and were candidates for major surgery. We compared two different pharmacological approaches, *i.e.,* morphine plus bupivacaine (30 patients, Group A)*versus*  morphine alone (30 patients, Group B). Each medication was administered by means of a thoracic epidural catheter for the control of postoperative pain. In the postoperative course we recorded every 6 hours peristaltic activity. We also noted morbidity (pneumonia, wound sepsis) and mortality. Effective peristalsis was present in all patients in Group A within the first six postoperative hours; in Group B, after 30 hours. Six patients in Group A had bowel motions in the first postoperative day, 11 in the second day, 10 in the third day and 3 in fourth day, while in Group B none in the first day, two in the second, 7 in the third, 15 in the fourth, and 6 in the fifth: the difference between the two groups was significant (*P*<0.05 in 1st, 2nd, 4th and 5th days). Pneumonia occurred in 2 patients of Group A, and in 10 of Group B (*P*<0.05).

We conclude that epidural analgesia with morphine plus bupivacaine allowed a move rapid return to normal gut activity and early enteral nutrition compared with epidural analgesia with morphine alone.


*HPB Surgery*, 2000, Vol. 11, pp. 393-399
Reprints available directly from the publisher
Photocopying permitted by license only

© 2000 OPA (Overseas Publishers Association) N.V.
Published by license under
the Harwood Academic Publishers imprint,
part of The Gordon and Breach Publishing Group.
Printed in Malaysia.

### Morphine Plus Bupivacaine *Vs.* Morphine Peridural Analgesia in Abdominal Surgery: The Effects on Postoperative Course in Major Hepatobiliary Surgery

G. BARZOI[a], S. CARLUCCIO[a], B. BIANCHI[a], S. VASSIA[b], G. COLUCCI[b] and G. L. MANGIANTE[b,*]

[a] *Institute of Anesthesiology and Intensive Care, University of Verona;* [b] *Department of Surgery, University of Verona, Italy*

*(Received 7 January 1998; In final form 18 July 1998)*

**Anaesthesia and surgical procedures lead to a reduction of intestinal motility, and opioids may produce a postoperative ileus, that might delay postoperative feeding. The aim of this prospective randomised study is to test whether or not different kinds of epidural analgesia (Group A: morphine 0.00 17 mg/kg/h and bupivacaine 0.125% - 0.058 mg/kg/h; Group B: morphine alone 0.035 mg/kg/12h in the postoperative period) allow earlier postoperative enteral feeding, enhance intestinal motility a passage of flatus and help avoid complications, such as nausea, vomiting, ileus, diarrhoea, pneumonia or other infective diseases. We included in the study 60 patients (28 males and 32 females) with a mean age of 61.2 years (range 50–70) and with an ASA score of 2 or 3. All patients had hepato-biliary-pancreatic neoplasm and were candidates for major surgery. We compared two different pharmacological approaches, *i.e.*, morphine plus bupivacaine (30 patients, Group A) *versus* morphine alone (30 patients, Group B). Each medication was administered by means of a thoracic epidural catheter for the control of postoperative pain. In the postoperative course we recorded every 6 hours peristaltic activity. We also noted morbidity (pneumonia, wound sepsis) and mortality. Effective peristalsis was present in all patients in Group A within the first six postoperative hours; in Group B, after 30 hours. Six patients in Group A had bowel motions in the first postoperative day, 11 in the second day, 10 in the third day and 3 in fourth day, while in Group B none in the first day, two in the second, 7 in the third, 15 in the fourth, and 6 in the fifth: the difference between the two groups was significant ($p < 0.05$ in 1st, 2nd, 4th and 5th days). Pneumonia occurred in 2 patients of Group A, and in 10 of Group B ($p < 0.05$).**

**We conclude that epidural analgesia with morphine plus bupivacaine allowed a move rapid return to normal gut activity and early enteral nutrition compared with epidural analgesia with morphine alone.**

*Keywords:* Peridural analgesia, postoperative pain relief, hepato-biliary surgery, early enteral nutrition

### INTRODUCTION

General anaesthesia (GA) and surgical procedures lead to a reduction of intestinal motility, especially gastric and rectal [1], opioids also produce postoperative ileus [2], that prevents early enteral nutrition (EEN) and a proper

*Address for correspondence: Department of Surgery, University of Verona, *Via* delle Menegone, 10, 37134-Verona, Italy.

intake. Feeding patients by enteral nutrition or by mouth as soon as possible seems to reduce the incidence of septic complications as well as costs of hospital stay. The higher infection rate of total parenteral nutrition (TPN) may arise from the vascular accesses necessary for its performance, but bacterial translocation from a fasting intestine can also lead to infections, such as pneumonia [3,4]. On the other hand, after surgical procedures, EEN often causes gastrointestinal discomfort, such as nausea, vomiting and diarrhoea. These complications may cause enteral nutrition to be stopped.

The aim of the present study is to test if a particular kind of peridural analgesia can give advantages in terms of faster return of gut activity and thus of earlier feeding of patients.

We compared two different pharmacological approaches *i.e.,* morphine plus bupivacaine *versus* morphine alone, for (A) effect on gastro-intestinal function; (B) intestinal transit; (C) morbidity (mostly represented by respiratory complications); (D) mortality. Each medication was administered through a thoracic epidural catheter inserted for the control of postoperative pain in patients who underwent major abdominal surgery for hepato-bilary diseases.

### MATERIALS AND METHODS

In a prospective randomised trial we enrolled 60 patients; 28 were male their mean age was 61.2 (range 50–70), the American Society of Anaesthesiology (ASA) score was 2–3. All the patients were affected by hepato-biliary neoplastic diseases and were candidates for major surgery. Antibiotic prophylaxis was administered. Patients with contraindications to epidural anaesthesia or with specific indications for mechanical ventilation (respiratory failure, oedema, and atelectasis) were excluded from the present study. The enrolling criteria are shown in Table I.

They were randomised to receive either epidural morphine plus bupivacaine (Group A: 30 patients), or epidural morphine alone (Group B: 30 patients). (Tabs. II and III)

TABLE I Criteria of patients enrollement

| Inclusion criteria |
|---|
| A–age between 50 and 70 |
| B–ASA score: 2–3 |
| C–major abdominal surgery for hepato-biliary neoplastic |
| D–antibiotic prophylaxis with Clindamycin 600 mg x 3/day and Gentamycin 80 mg x 3/day one hour before operation and for the following 72 hour |
| E–pre- and postoperative physiotherapy |
| F–non-smokers |
| G–no irritable bowel syndrome (diseases associated with abnormal bowel habits) |
| H–no reflux disease or hiatal hernia |
| I–Surgical sympathectomy was a retrospective criterion of exclusion |

TABLE II Characteristics of the patients

| Characteristics of patients | Group A | Group B | Group A *vs* B |
|---|---|---|---|
| no of patients | 30 | 30 | |
| age: mean | 61.7 | 60.6 | $p$ = n.s. |
| Median | 62.0 | 59.5 | $p$ = n.s. |
| sex (M/F) | 12/18 | 16/14 | $p$ = n.s. |
| weight: mean | 63.4 | 63.1 | $p$ = n.s. |
| weight: median | 63.0 | 63.0 | |
| ASA 2 | 18 | 16 | $p$ = n.s. |
| ASA 3 | 12 | 14 | $p$ = n.s. |

TABLE III Associated diseases of the enrolled patients

| Associated diseases | Group A | Group B |
|---|---|---|
| Ischemic heart disease | 7 | 5 |
| Left ventricular hypertrophy | 5 | 6 |
| Peripheral vascular disease | 4 | 3 |
| COPD/emphysema | 13 | 11 |
| Insulin dependent diabetes mellitus | 4 | 5 |
| Peptic ulcer | 7 | 4 |
| Chronic pancreatitis | 2 | 0 |
| Cirrhosis | 9 | 11 |

TABLE IV Hepato-biliary procedures performed on the patients

| Surgical procedures | Group A | Group B |
|---|---|---|
| Hepatic neoplasm | 4 RH, 3 LH, 5 LLL, 3BS, 10 WR | 5 RL, 5LH, 7LLL, 9 WR |
| Biliary neoplasm | 2 RH + BR, 2 LH + BR, 1 IHJ | 1 RL + BR, 3 IHJ |

RH: right hepatectomy, LH: left hepatectomy, LLL: excision segments 2–3, BS: bisegmentectomy, WR: wedge resection, BR: biliary confluence resection, IHJ: intrahepatoductal-jejunoanastomosis.

The operations performed are summarised in Table IV.

On the day of operation an epidural catheter was inserted at T6–T7 level and was inserted 3 cm into epidural space; a Mepivacaine and Fentanyl mixture was administered through this catheter. After 20 minutes the level of anaesthesia was tested by pinprick. All the patients were subsequently submitted to oro-tracheal intubation and to mechanical ventilation after induction with Thiopental and Vecuronium; maintenance of anaesthesia was with Isofluorane 0.3–0.5%.

One hour before the end of operation we started postoperative analgesic therapy: the same treatment was continued for 36 hours. Group A patients were administered non-stop postoperative epidural analgesia with morphine (0.0017 mg/kg/h) and bupivacaine 0.125% (0.058 mg/kg/h); group B patients, were given epidural analgesia with a morphine bolus every 12 hours (0.035-mg/kg/12 h). The treatment was administered by some of the authors (GB, SC, BB) and the results were analysed by the others (GM, SV, and GC), who were unaware of the kind of analgesic treatment, which had been used.

Postoperatively, we tested several parameters every 6 hours for 36 hours altogether. The analgesic effectiveness was assessed according to the Scott–Huskisson [5] visual analogic pain scores (VAS) from 0 to 10 [9] with score 1 = no pain and score 10 = maximum bearable pain; this evaluation was made with the patient at rest, during exercise and during expectoration. The cardio-circulatory and respiratory stability was evaluated by monitoring the average arterial pressure, the heart rate and the respiratory rate with the patients both standing and lying. Finally we monitored the onset of peristalsis by listening for bowel sounds. By prolonging the observation period up to one week we evaluated the gut transit time. Alteration of mental status was assessed with the classification proposed in the Aldrete score: patient awake = 2, drowsy but arousable = 1, unresponsive = 0. Postoperatively, we used no drugs that affect peristalsis. We started enteral nutrition in all patients within the first 48 hours through a naso-jejunal tube positioned during operation: EEN flow was the same in all patients 25 ml/h on day one, 45 ml/h on day two, and 85 ml/h on the day three. Parenteral nutritional support was also performed by until reaching a sufficient caloric intake. The statistical significance of the continuous variables was calculated by analysis of variances. Visual analogic pain scores were compared with Mann–Whitney test. Statistical analysis included the $\chi^2$ test for categorical data. $P$-values of less than 0.05 were considered statistically significant.

### RESULTS

The patients in the two groups were homogeneous for age, sex, weight and clinical characteristics.

All the enlisted patients were included in the study.

The level of analgesia reached was between T3 and L4.

VAS at rest was $\leq 3$ with a statistically significant difference only between the two groups in the first survey, with better analgesia in Group A. During exercise and expectoration the analgesic control was not so effective, although there were no statistically significant differences.

No significant changes in mean arterial pressure, heart rate and respiratory rate were recorded with patients standing and lying.

Bowel sounds were already present in all the patients in group A at the first data survey which was made 6 hours postoperatively. In group B, at the first survey bowel sounds were present in 2/30 (6.7%) patients. Over the next 24 hours peristalsis appeared in other 16/30 (53.3%) patients; after 30 hours it appeared in the remaining patients (Tab. V).

Alvus occurred in the following sequence: in group A, in 6/30 (20%) patients on day one, in 11/30 (36.7%) patients on day two, in 10/30 (33.3%) on day three, and in the remaining 3/30 (10%) on day fourth; in group B, 2/30 (6.7%) patients were canalised on day two, 7/30 (23.3%) on day third, 15/30 (50%) on day fourth and the rest on day fifth. The first 2 days we had statistically significant differences between the 2 groups, with $p = 0.031$ on day one and $p = 0.012$ on day (Tab. VI).

We found no alterations of mental status; all the patients were in fact assigned a score equivalent to an Aldrete score of 2.

Nausea appeared in 3/30 (10%) patients in group A and in 2/30 (6.7%) in group B; in 1/30 (3.3%) patient in each group isolated episodes of vomiting occurred during the first 18 hours.

The comparison between the two groups showed no significant differences in terms of mortality: two deaths occurred, one in each group, caused by generalised sepsis subsequent to pneumonia; in the patients in the group A both blood and sputum cultures were positive

TABLE V Resumption time of peristaltic activity

| Appearance of peristalsis in the postoperative period | Group A | Group B |
|---|---|---|
| 6th hour | 30 | 2 |
| 12th hour | 0 | 2 |
| 18th hour | 0 | 5 |
| 24th hour | 0 | 9 |
| 30th hour | 0 | 12 |

TABLE VI Return time to normal intestinal transit

| Time for return of normal gut activity | | | | | |
|---|---|---|---|---|---|
| Day after surgery | 1st | 2nd | 3rd | 4th | 5th |
| Group A pts | 6 | 11 | 10 | 3 | 0 |
| Group B pts | 0 | 2 | 7 | 15 | 6 |
| stat significance | $p = 0.031$ | $p = 0.012$ | $p =$ n.s. | $p = 0.002$ | $p = 0.031$ |

for *Candida Albicans,* while group B sputum cultures were negative and blood cultures became positive for *Staphilococcus Aureus.*

No cases of wound infection occurred.

During the first week after operation, in 8/30 (26.7%) patients of group A atelectatic areas were noticed on X-ray, while this complication happened in 18/30 (60%) patients of group B. We found pneumonia present in 2/30 (6.7%) patients of group A and in 10/30 (33.3%) patients of group B; the difference was statistically significant ($p = 0.024$).

### DISCUSSION

Our study compares two different pharmacological approaches to control postoperative pain *via* epidural catheterization, by evaluating their role in intestinal transit. We tried to determine whether or not this postoperative treatment could be correlated with the occurrence of postoperative infections as well. Experimental and clinical trials prove that after surgery the early introduction of enteral nutrition reduces the incidence of postoperative complications, because it minimises the catabolic response to biological damage, preserves intestinal bacterial flora, and maintains integrity of the gastrointestinal mucosa. In this way EEN helps to reduce the incidence of infections, sepsis and mortality subsequent to bacterial translocation [4]. We often see postoperative ileus following major abdominal surgery. This dysfunction represents a drawback to enteral nutrition in critically ill patients. Diarrhoea, vomiting and abdominal distension often hinder initial administration and subsequent tolerance [6]. Among possible causes we have to consider traumatic manipulation of viscera, electrolyte changes, anaemia, malnutrition and prolonged immobility of patients.

The results of studies on gastrointestinal motility in the postoperative period in both animals and humans are contradictory: a lot of devices have been employed to demonstrate the onset of enteric motility, such as electrodes placed into the gut wall [7], test meal [1], small bowel manometry [8] *etc.* We think that the real clinical evidence of the onset of effective enteric motility is the recommencing of intestinal transit [9].

The sympathetic-adrenergic reflex plays an important role in the patho-physiology of postoperative ileus. Such reflex is carried partly by afferent pain fibers and plays a role in curtailing blood flow and in reducing peristaltic activity. Control of postoperative pain gives thus an important contribution to the return of gut activity [2, 10].

Postoperative ileus is partially reversed by splanchnicectomy: this action demonstrates the role of the sympathetic nervous system in postoperative ileus. The stimulation of the sympathetic system decreases motility, while the parasympathetic system increases it [9].

The association of surgery and stress results in an increase of vaso-active hormones, such as catehcolamines and vasopressin in the bloodstream, which decrease bowel motility [9]. Peristalsis may be enhanced or inhibited by the autonomous nervous system; both parasympathetic and orthosympathetic nerve fibers end in Auerbach myoenteric plexus [9]. The parasympathetic system acts as an exciter while the sympathetic system acts as an inhibitor. This kind of innervation exercises an overall control on intestinal musculature by modulating its activity, but it is not necessary for activity occur because it is of myogenic origin [11]. Autonomous innervation is therefore likely to regulate and modulate intrinsic intestinal activity.

Intestinal parasympathetic branches originate in the vagus, while sympathetic innervation is carried by nervous fibers such as the splanchnic nerves that contain both afferent and efferent visceral fibers.

Stimulation of intra-abdominal receptors caused by surgical manipulations seems to trigger off spinal reflexes that enhance sympathetic activity through afferent fibers of the splanchnic nerves: this action results in inhibition of peristaltic activity [11].

An anaesthetic epidural block at the level of T5 – L1 causes denervation of abdominal viscera with a block of the splanchnic nerves; such a sympathetic block favours peristaltic activity because of the predominance of parasympathetic tone [12].

The analgesic effect of morphine on postoperative pain is well known [13]: the drug reduces enteric propulsive waves by blocking the action of cholinergic, triptaminergic and encephalinergic receptors on the myoenteric plexus [14]. We have to reduce the dosage of morphine into the epidural space because respiratory depression occurs after epidural administration of 2 mg of the drug, which is due to epidural spreading [15]. The reduction of morphine is allowed because of the synergistic action of the local anaesthetic.

In our series we found out that, in patients who had epidural analgesia with morphine plus bupivacaine, the return of gut activity occurred earlier than in those who had been given only morphine [16 – 19].

We think that the lower incidence of pneumonia in Group A is due to the earlier return of gastrointestinal motility and thus to a lower incidence of bacterial translocation with subsequent reduction of infectious complications, improved expectation might also have made a contribution.

### CONCLUSIONS

Our results stress the importance of the effective control of postoperative pain on the onset of peristaltic activity after abdominal surgery. The reduction of the dosage of morphine allowed by the use of local anaesthetic *via* epidural administration may play an important role in reducing postoperative pain and at the same time in the restoration of effective peristaltic waves so as to allow earlier enteral feeding. Nowadays, the gold standard in the postoperative period should be to prevent septic complications through EEN, pain relief, and quick restoration of normal intestinal transit.

### References

[1] Maes, B. D., Ghoos, Y. F., Geypens, B. J., Hiele, H. L. and Rutgeerts, P. J. (1996). Relation between gastric emptying rate and rate of intraluminal lipolysis. *Gut*, **38**, 23 – 27.

[2] Thoren, T. and Wattwil, M. (1988). Effects on gastric emptying of thoracic epidural analgesia with morphine or bupivacaine. *Anesth. Analg.*, **67**, 687 – 94.

[3] Aranow, J. S. and Fink, M. P. (1996). Determinants of intestinal barrier failure in critical illness. *Br. J. Anesth.*, **77**, 71 – 81.

[4] Moore, F., Feliciano, D. V., Andrassy, R. J., Mc Ardle, A. H., Booth, F. V., Morgenstein-Wagner, T. B., Kellum, J. M., Welling, R. E. and Moore, E. E. (1992). Early enteral feeding, compared with parenteral, reduces postoperative septic complications. *Ann. Surg.*, **216**, 172 – 83.

[5] Scott, J. and Huskisson, E. G. (1976). Graphic representation of pain. *Pain*, **2**, 175 – 84.

[6] Heyland, D., Cook, D. J., Winder, B., Brylowski, L., Van de Mark, H. and Guyatt, G. (1995). Enteral nutrition in the critically ill patient: a prospective survey, *Crit. Care Med.*, **23**, 1055 – 60.

[7] Hotokezaka, M., Mentis, E. P., Patel, S. P., Combs, M. J., Teates, C. D. and Schirmer, B. D. (1997). Recovery of gastrointestinal tract motility and myoelectric activity change after abdominal surgery. *Arch. Surg.*, **132**, 410 – 417.

[8] Benson, M. J., Roberts, J. P., Wingate, D. L., Rogers, J., Deeks, J. J., Castillo, F. D. and Williams, N. S. (1994). Small bowel motility following amjor intyrta-abdominal surgery: The effects of opiates and rectal cisapride. *Gastroenterology*, **106**, 924 – 936.

[9] Livingston, E. H. and Passaro, E. P. (1990). Postoperative ileus. *Dig. Dis. Sc.*, **35**(1) 121 – 132.

[10] Scheinin, B., Asantila, R. and Orko, R. (1987). The effect of bupivacaine and morphine on pain and bowel function after colonic surgery. *Acta Anaesthesiol Scand.*, **31**, 161 – 4.

[11] Moss, J. and Craigo, P. A. (1994). The Autonomic Nervous System. In: Miller, R. L., *Anesthesia. Churchill Livingstone*, New York, **1**, 523 – 575.

[12] Haberer, J. P. (1991). Peridural Anesthesia in Gauthier-Lafaye P: Manuale di Anestesia loco-regionale. *Masson Paris*, **12**, 201 – 245.

[13] Harrison, D. M., Sinatra, R., Morgese, L. and Chung, S. H. (1988). Epidural narcotic and patient- controlled analgesia for post-caesarean section pain relief. *Anesthesiology*, **68**, 454 – 457.

[14] Burks, T. F. (1976). Gastrointestinal Pharmacology. *Annu. Rev. Pharmacol Toxicol.*, **16**, 15 – 31.

[15] Gourlay, G. K., Cherry, D. A. and Cousins, M. J. (1985). Cephalad migration morphine in CSF following lumbar epidural administration patients with cancer pain. *Pain*, **23**, 317 – 326.

[16] Dahl, J. B., Hjortso, N. C., Stage, J. C., Hansen, B. L., Moiniche, N. S., Damgaard, B. and Kehlet, H. (1994). Effects of combined perioperative epidural bupivacaine and morphine, ibuprofen, and incisional bupivacaine on postoperative pain, pulmonary, and endocrine-metabolic function after minilaparotomy cholecystectomy. *Reg. Anesth.*, **19**, 199–205.
[17] Moiniche, S., Dahl, J. B., Rosenberg, J. and Kehlet, H. (1994). Colonic resection with early discharge after combined subarachnoid-epidural analgesia, preoperative glucocorticoids, and early postoperative mobilization and feeding in a pulmonary high-risk patient. *Reg. Anesth.*, **19**, 352–6.
[18] Thorn, S. E., Wattwil, M. and Kallander, A. (1994). Effects of epidural morphine and epidural bupivacaine on gastroduodenal motility during the fasted state and after food intake. *Acta Anaesthesiol Scand.*, **38**, 57–62.

### COMMENTS

This is a study on the postoperative management after major hepatobiliary surgery. The authors have focused on bowel function and shown in a randomized trial that this can be achieved by a combination of Morphine and Bupivacaine better than with Morphine alone in epidural administration. The importance of enteral administration of nutrients after surgery is especially important after hepatobiliary surgery. One reason is the increased risk for bacterial translocation after liver resection in a patient with a non functioning bowel Ref. [1]. Furthermore the stimulation of liver regeneration and restoration of liver function are also dependent on factors released from the gastrointestinal tract and in this respect an early return of gut function and start of enteral feeding are crucial [2]. With the proper management in the early post-operative period, as it is shown in this randomized trial, an early return of bowel function can be achieved allowing early post-operative enteral nutrition to be resumed. This will hopefully lead to reduced problems with post-operative infections and organ failure.

### *References*

[1] Bacterial translocation after major hepatectomy in patients and rats. Wang, X. D., Andersson, R., Soltesz, V. and Bengmark, S. (1992). *Annals of Surgery*, **127**, 1101–1106.
[2] La Brecque, D., Liver regeneration: a picture emerges from the puzzle. *The American Journal of Gastroenterology*.

Prof. B. W. Jeppsson

Department of Surgery

Malmö University Hospital

Malmö

S-205 02

Sweden

